# Functional capacity and dual-task cost in the institutionalized older adults, both affected and unaffected by mild cognitive impairment

**DOI:** 10.1186/s11556-021-00270-0

**Published:** 2021-07-12

**Authors:** Marek Zak, Szymon Krupnik, Waldemar Brola, Dorota Rebak, Tomasz Sikorski, Frederic Dutheil, Jaroslaw Andrychowski, Daniel Courteix

**Affiliations:** 1 The Institute of Health Sciences, Collegium Medicum, The Jan Kochanowski University, ul. Zeromskiego 5, 25-369 Kielce, Poland; 2 Symmetry, Medical Rehabilitation Centre, Sosnowiec, Poland; 3 CNRS, LaPSCo, Physiological and Psychosocial Stress, Université Clermont Auvergne, Clermont- Ferrand, France; 4 Departement de Médecine du travail, Clermont-Ferrand University Hospital, G. Montpied Hospital, Clermont-Ferrand, France; 5 Laboratory of the Metabolic Adaptations to Exercise under Physiological and Pathological Conditions (AME2P - EA 3533), University Clermont Auvergne, Clermont-Ferrand, France

**Keywords:** Older adults, MCI, Dual-task, Institutionalised care, Public health

## Abstract

**Background:**

Mild cognitive impairment (MCI) affects 10–20% of the individuals over the age of 65; this proportion being higher in the institutional care facilities than within a general population.

**Aim:**

To assess whether dual-task cost in the individuals affected by MCI depends exclusively on gait, or possibly some other functional capacity components might also come into play, as compared to the healthy controls also remaining in the institutional care.

**Methods:**

The study was conducted in five nursing facilities, involving 88 subjects in total, i.e. 44 subjects affected by MCI (mean age of 83.8 years; 34 women (77.3%) and 10 men (22.7%), and 44 healthy controls (mean age 81.67 years; 38 women (84.4%) and 7 men (15.6%). Cognitive functions were assessed through Mini–Mental State Examination (MMSE), while gait by Timed Up and Go Test (TUGT). Gait speed was calculated by the 10 Meter Walk Test, and the fear of falling with the Falls Efficacy Scale International. Dual tasks were assessed by TUGT_MAN_ (Timed Up and Go Test Manual) and TUG_COG_ (Timed Up and Go Test Cognitive). Dual Task Cost (DTC) of TUGT_MAN_ and TUGT_COG_ was established. Statistical analyses were completed with STATISTICA Package v. 10.

**Results:**

Individuals affected by MCI differed significantly from the unaffected ones with regard to their gait test results, when assigned a single-task activity, and dual-task activities, as well as in the gait speed. Dual Task Cost Manual (DTC_MAN_) in the MCI group was significantly higher, as compared to the subjects unaffected by MCI.

Around 25% of the variance of DTC_MAN_ result regarding the MCI group was accounted for by gait performance in the single-task conditions (TUGT). In the case of Dual Task Cost Cognitive (DTC_COG_), this value equalled to approx. 10%. A 1% change in DTC_MAN_ corresponded to approx. 0.5 s change in TUGT, whereas a 1% change in DTC_COG_ entailed approx. 0.35 s change in TUGT walking time.

**Conclusion:**

Individual functional capacity affected the dual-task performance, especially the motor-motor tasks. Dual-task cost in the subjects affected by MCI was significantly reduced, being more dependent on the gait speed in the motor-motor tasks, which entailed visual memory, than in the motor-cognitive tasks.

## Introduction

Mild cognitive impairment (MCI) is defined as cognitive impairment related to individual age- and education-related abilities, which has no bearing on executing the activities of daily living (ADLs) [1]. MCI affects 10–20% of the individuals over the age of 65 [2, 3]. Cahill et al. demonstrated that approx. 89% of institutionalized persons experienced mild to severe symptoms of dementia [4]. There is a correlation between cognitive function and gait, since – as demonstrated by Srygley et al. – gait is not a fully automatic activity. In fact, it also entails a cognitive component [5]. Montero-Odasso et al. demonstrated that individuals affected by MCI exhibited reduced stride time variability, while dual tasking [6]. In their study, poor executive functions, as well as working memory were associated with low gait speed.

Lee et al. demonstrated that Dual-task cost (DTC) in the individuals affected by MCI was greater with regard to regular gait, compared to those unaffected by this impairment, priority gait (two-fold greater), and cognitive prioritisation gait (three-fold greater) [7]. Also, the individuals exposed to Motoric Cognitive Risk (MCR) syndrome, construed as subjective cognitive complaints, a predictor of dementia, are characterised by reduced gait speed, information processing speed, and executive functions, without delayed free recall memory [8] when assigned a dual-task regimen [9]. Regardless of these reports, it has not been established beyond a reasonable doubt whether DTC in the individuals affected by MCI depends solely on gait, or whether other functional capacity components might also be entailed.

### Purpose

The present study aimed therefore to establish conclusively whether DTC in the individuals affected by MCI depends exclusively on gait, or possibly some other functional capacity components might also come into play, thus making this relationship far more complex in terms of causal factors, as compared to the healthy controls unaffected by MCI. It was assumed that individuals affected by MCI had higher DTC in Timed Up and Go Test Manual (TUGT_MAN_) vs. Timed Up and Go Test Cognitive (TUGT_COG_)_._

## Methods

### Patients and methods

The study was conducted in five nursing facilities, involving 88 subjects in total, i.e. 44 subjects with MCI (mean age of 83.8 years; 34 women (77.3%) and 10 men (22.7%)) and 44 healthy controls unaffected by MCI (mean age 81.67 years; 38 women (84.4%) and 7 men (15.6%)).

The following inclusion criteria were applied: age (> 65 years), absence of neurological or auditory deficits (i.e. a history of stroke with visible functional deficits of the locomotor system, sciatica, cerebral injuries), regular use of glasses with a power not exceeding 4 dioptres, and a Mini–Mental State Examination (MMSE) score of less than 24 points, although not less than 18, Barthel scale < 40 points [10]. These criteria were supplemented by those proposed in the study by Winblad et al. [2]. Furthermore, any elderly individuals who were found unable to complete any of the functional ability tests, were at that point pronounced non-eligible for further attendance in the study protocol.

Representativeness was calculated using a sample-size calculator:

(http://www.nss.gov.au/nss/home.nsf/pages/Sample+size+calculator).

For a population of 500,000 (a proportion of 0.5, and a standard error of 5.1), in conjunction with the 1.5 odds ratio (OR) for a decrease in the gait speed in the group affected by MCI, the determined representative population sample size was 44 subjects (MCI group), and 44 subjects unaffected by MCI (healthy controls group). The subjects were recruited by the onsite specialists employed in each nursing facility. Sociodemographic data of the individuals affected by MCI were acquired from their medical records. In the case of healthy controls, the data were acquired directly from the subjects. The variables addressing individual functional ability were presented as the data characterising both the study and the control groups. The testing protocol was executed by a trained physiotherapist boasting a minimum 2-year hands-on working experience with the elderly patients, and prior experience in pursuing academic research projects.

Prior to the actual commencement of the study protocol, the subjects were verbally introduced to its practical specifics. A general presentation was made, whereas the key points of the testing procedure were shown in the form of pictograms. All applicable constraints for the execution of the tests were fully compliant with the breakdown provided further below.

At the First Session, pertinent sociodemographic data were collected, followed by the assessment of individual cognitive abilities against the MMSE. The score acquired in the MMSE test was a qualifier for further attendance in the study protocol. In practical terms, if an individual failed to score within the 18–23 points range, he was pronounced non-eligible for further participation in the study protocol.

Subsequently, Timed Up and Go Test (TUGT), TUGT_MAN,_ and TUGT_COG_ were completed. Single- and dual-task tests were carried out 3 times, whereas the mean score was ultimately recorded.

The Second Session comprised the 10 Meter Walk Test (10MWT) tests, both in the regular, and the fastest version, Strength of Lower Limbs and Fear of Falling (FOF), were also assessed.

The Third Session comprised the execution of 3 Single Leg Stance Open Eyes (SLS OP) and Single Leg Stance Closed Eyes (SLS CL) tests.

The examination of participants remaining in institutional care lasted 1.5 h in total, 30 min per each session.

Healthy subjects were tested in their own place of residence, in full compliance with the procedure outlined further above.

All participants were granted a 3–5 min period of rest between respective tests assessing individual functional performance. Subjects were assessed 2 h after a meal, in the morning hours.

### Cognitive functions

Cognitive functions were assessed through MMSE, also known as the Folstein test, which facilitates assessment of five cognitive functions, i.e. orientation, memory, concentration, language, and constructional praxis [11].

The corresponding criterion for inclusion into the study group was the score ranging 18–23 points, as per applicable guidelines in the literature on the subject, indicative of mild cognitive impairment [11]. The test was completed by a trained physiotherapist, under the same conditions for each subject.

### Gait performance assessment under the single- and dual-task conditions

The version of TUGT test proposed by Podsiadlo was used to evaluate gait performance under the single-task conditions [12]. The subjects were advised that the correct way to perform the test was to stand up from a chair and then walk a 3-m distance behind the line, then turn around, walk all the way back, and sit down in the chair. The walk should be completed at the fastest possible pace deemed safe by the test subject. Under the dual-task conditions, gait performance was assessed by means of yet another version of the TUGT test, i.e. the one modified in line with Shumway-Cook and Wollacotte [13]. Dual-task activities were divided into the two types, i.e. motor-motor tasks (manual TUGT – TUGT_MAN_) and motor-cognitive tasks (cognitive TUGT – TUGT_COG_). Both types of activities were performed following verbal and non-verbal instructions given by a physiotherapist.

During the TUGT_MAN_ test, the subjects were asked to perform the same task as the TUGT test, while holding a beaker filled up with water in their dominant hand, whereas with regard to the TUGT_COG_ test, the subjects were supposed to incrementally count down by 7, starting off with a randomly selected number between 20 and 100, while walking. During either type of the dual-task test (TUGT_MAN_ and TUGT_COG_), a physiotherapist did not indicate which task had a higher priority. All timings were taken with the aid of a mobile phone (Apple iPhone 6 – model A1586), and a built-in stopwatch application with an accuracy of 0.01 s. A drop in the performance of dual-task activities was considered clinically significant, when the DTC, expressed as the relative difference between the gait speed under the dual-task conditions and that under the single-task ones, and then calculated in line with the formula given by Bock, was higher than 15% [14]. In conformity with the algorithm proposed by Bock, an average DTC for healthy elderly individuals amounted to 15%, having effectively been calculated out of 13 different dual-task activities; the same criterion was consequently assumed in this study protocol [14].

The gait speed corresponding to either the single-task, or the dual-task conditions was determined by dividing the distance covered during the TUGT, TUGT_MAN_ and TUGT_COG_ tests by the respective time it took to do so.

These tests were selected in view of being comprised of four essential components directly aiding the research effort, as well as offering the possibility of assessing a scope of ADLs:

1. Exclusion of the learning effect (TUGT test is performed first).

2. Very high test reproducibility [15].

3. An opportunity to assess short-term memory and metastability of attention during complex activity.

4. This test assesses the ability to stand up, walk, turn around, and sit down, i.e. an essential component in an individual pursuit of the ADLs.

### Gait speed

Gait speed was calculated with the aid of the 10MWT, whereby the subject had to walk a distance of 10 m. In line with the test methodology, the first and the final metre were excluded from the measurements [16]. Additionally, the two gait speeds were measured, i.e. normal, everyday speed, and the fastest speed the subject felt comfortable with.

## FOF

The fear of falling was assessed by means of the Falls Efficacy Scale International Version (FES-I) [17, 18]. This scale consists of 16 components, each of which is scored 1–4 points, whereby a minimum score of 16 points indicates no fear of falling, whereas a maximum score of 64 points indicates high fear of falling [17, 18].

### Strength of the lower limbs

The strength of the lower limbs was assessed using the 30-s chair stand test (30sChS), pursued in the way described by Jones and Rikli [19]. A physiotherapist counted aloud the number of repetitions, yet never verbally encouraged the subject to do any additional repetitions.

### Aerobic endurance

Aerobic endurance was assessed with the aid of a 2-Minute Step (2-Min. Step) test. In line with the methods proposed by Rikli and Jones [20], the subjects pursued a stationary walk for two minutes, while lifting their knees halfway between the patella and the iliac crest in a standing position. The outcome of the test was determined by the number of right limb raises above the line drawn on the wall, for each subject respectively.

### Static balance

The single leg stance (SLS - Single Leg Stance) test was performed to assess static balance. The test was carried out under two different conditions, i.e. with the eyes open SLS OP, and then with the eyes closed SLS CL. The subject was to maintain balance while standing on the dominant leg [21]. The test ends when the foot touches the ground and the result is the actual duration (in seconds) of maintaining balance by the test subject.

### Statistical analysis

Statistical data were processed with the aid of the STATISTICA 10 software package for Windows. Descriptive statistics were also completed. Normal/Gaussian distributions were calculated by means of the Shapiro–Wilk test (*n* < 100). Spearman’s rank correlation coefficient was calculated to determine the level of interdependence of variables. The Mann–Whitney U test was pursued for quantitative variables. DTC (%) = 100*(single task score - dual task score)/single-task score [14].

An adjusted multi-nominal regression model was developed, with a view to indicating the effect of the independent variable TUGT on the dependent variable Dual Task Cost Manual (DTC_MAN_) in the subjects affected by MCI. The adjusted model was standardised against the select anthropometric data (i.e. sex, age, height, weight, and body mass index). The alpha significance level was set at = 0.05.

## Results

### Intergroup differences in the DTC

Statistical analysis indicated that MCI subjects had a higher motor - motor cost (mean difference between the two groups 8.73 of DTC, the percentage differences between the two groups 104.3%, *p* < 0.01) and a lower motor-cognitive cost (mean difference between the two groups 13.2 of DTC, the percentage differences between the two groups 34.11%, *p* < 0.05), as compared to the control group. Significantly fewer subjects affected by MCI had a dual-task, motor-motor and cognitive-motor cost, as compared to the control group, by 63.5 and 14.4%, respectively. The MCI subjects differed in gait speed in TUGT, TUGT_MAN_, TUGT_COG_ gait tests by 58.8, 86.7, and 57.7%.

Baseline characteristics of the study subjects are comprised in Table 1. All test results are comprised in Table 2.

**Table 1:** Baseline characteristics of the study subjects

Variable	MCI ***N*** = 44	Healthy Group ***N*** = 44	***p***
**Demographic**			
Gender (female/male%)	77/23	84/16	0.55*
Age (years),Mean, SD	83.82 (8.5)	81.67 (3.5)	0.076*
Body weight (kg), Mean, SD	63.90 (14.9)	71.83 (8)	0.006*
Body height (cm), Mean, SD	161.18 (9)	161.50 (7)	0.971*
Body mass index (kg/m^2^) Mean, SD	24.31 (4.8)	27.66 (3.6)	0.057*
**Education**			
Primary (%)	29.5	23.8	0.091**
Secondary (%)	18.2	38.1	0.001**
University (%)	40.9	9.5	0.001**
Other (vocational training only) (%)	11.3	14.3	0.64**
**Marital status**			
Married (%)	15.9	14.3	0.43**
Unmarried (%)	11.4	4.8	0.041**
Divorced (%)	6.8	9.5	0.091**
Widowed (%)	65.9	71.4	0.14**
**Medication**			
Average p/day, Mean, SD	7 (5)	5.78 (4.7)	0.044*
< 4 medication p/day (%)	29.5	38.9	0.004**
> =4 medication p/day (%)	70.4	61.1	0.004**
**Concomitant diseases**			
1 (%)	47.7	47.6	0.514**
2 (%)	15.9	11.1	0.081**
> 2 (%)	36.3	41.3	0.003**
**Cognitive status**			
MMSE (pts.), Mean, SD	20.34 (7.7)	27.72 (3.1)	0.001*
**Gait Assessment**			
TUGT (s), Mean, SD	20.58 (8.0)	10.99 (4.6)	0.002*
TUGT_MAN_ (s), Mean, SD	24.1 (7.9)	11.91 (4.5)	0.001*
TUGT_COG_ (s), Mean, SD	25.62 (10.4)	14.74 (5.5)	0.001*
10MWT normal speed (m/s), Mean, SD	0.57 (0.2)	0.94 (0.3)	0.004*
10MWT fast speed (m/s), Mean, SD	0.62 (0.2)	1.1 (0.2)	0.005*
**FOF**			
FES-I, Mean, SD	33.1 (15.1)	36.28 (3.3)	0.77*
**Strength of Lower Limbs**			
30sChair Stand (n of stand ups), Mean, SD	9.06 (3.8)	9.22 (4.5)	0.817*
**Aerobic Endurance**			
2-Min Step (number of steps), Mean, SD	77.43 (42.5)	84.33 (44.7)	0.061*
**Static Balance**			
SLS OP (s)	2.52 (4.7)	4.04 (3.4)	0.119*
SLS CL (s)	2.02 (4.1)	2.7 (3.3)	0.288*
**Falls**			
No (%)	63.6	57.1	0.359*
1 (%)	36.4	28.6	0.359*

**Abbreviations:** SD-Standard Deviation, TUGT – Timed Up and Go, TUGT_MAN_ – Timed Up and Go Manual, TUGT_COG_ – Timed Up and Go Cognitive, 10MWT-10 Meter Walk Test, FOF – Fear of Fall, FES-I – Falls Efficacy Scale International version, 2Min Step – Two Minute Step test, SLS OP – Single Leg Stance Open Eyes, SLS CL – Single Leg Stance Closed Eyes

*Chi2, **U-Mann Whitney

**Table 2:** Intergroup differences in the dual-task cost

Variable	MCI Group ***N*** = 44	Healthy Group ***N*** = 44	p
**DTC**(%)			
DTC_MAN_, Mean, SD	17.1 (2.7)	8.37 (3.1)	0.002**
DTC_COG_, Mean, SD	25.5 (12.1)	38.7 (27.6)	0.049**
DTC_MAN_ **<** 15%	4.5	68	0.001*
DTC_COG_ < 15	2.3	16.7	0.007*
**Gait speed**(m/s)			
TUGT, _Mean, SD_	0.34 (0.2)	0.54 (0.5)	0.003**
TUGT_MAN, Mean, SD_	0.27 (0.2)	0.50 (0.4)	0.005**
TUGT_COG, Mean, SD_	0.26 (0.2)	0.41 (0.2)	0.003**
**Single vs TUG**_**MAN**_(m/s)			
TUGT vs TUGT_MAN, Mean, SD_	0.06 (0.1)	0.03 (0.1)	0.003**
TUGT vs TUGT_COG_ Mean, SD	0.07 (0.3)	0.13 (0.3)	0.416**
**Single task vs TUGT**_**MAN**_(n%)			
Slower gait speed	86.4	81.6	0.641*
Faster gait speed	13.6	18.4	0.511*
**Single task vs TUGT**_**COG**_(n%)			
Slower gait speed	90.9	94.5	0.071**
Faster gait speed	9.1	5.5	0.071**

**Abbreviations:** *Chi2, **U-Mann Whitney

### DTC

The DTC for dual-task conditions that entailed reverse counting (TUGT_COG_) was 8.4% higher than the cost of motor-motor tasks (TUGT_MAN_) in the MCI group, and 30.33% higher than in the case of the healthy controls unaffected by MCI.

All DTC results are comprised in Table 2.

### Single-task performance

There was a negative, statistically significant correlation between the single-task gait (TUGT) performance and the following factors: both normal and fast gait speed during the 10MWT, strength of the lower limbs (30sChS), static balance with both open and closed eyes, and TUGT_MAN_ and TUGT_COG_. The respective correlations were weak (SLS OP, SLS CL), moderate (10MWT – normal speed), strong (10MWT – fast speed, 30sChS), and very strong (TUGT_MAN_ and TUGT_COG_).

### Dual-task performance and functional outcomes in the MCI group

Dual-task gait was assessed by means of two tests, i.e. TUGT_MAN_ and TUGT_COG_. There was a negative, statistically significant correlation between the dual-task motor-motor gait performance (TUGT_MAN_) and the following factors: both normal and fast gait speed during the 10MWT, the strength of the lower limbs (30sChS), static balance with both open and closed eyes, and TUGT and TUGT_COG_. A positive, statistically significant correlation was observed with the FES-I. The respective correlations were weak (FES-I), moderate (10MWT – normal and fast speed, 30sChS, SLS OP, SLS CL), strong (30sChS), and very strong (TUGT and TUGT_COG_).

There was a negative, statistically significant correlation between the dual-task motor-cognitive gait performance (TUGT_COG_) and the following factors: both normal and fast gait speed during the 10MWT, the strength of the lower limbs (30sChS), static balance with both open and closed eyes, and TUGT and TUGT_MAN_. The respective correlations were weak (SLS OP), moderate (10MWT – normal and fast speed, 30sChS, SLS CL), strong (30sChS), and very strong (TUGT and TUGT_MAN_). Spearman’s rank correlation coefficient, functional assessment and Single- and Dual-Task variables are outlined in Table 3.

**Table 3:** Spearman’s rank correlation coefficient, functional assessment and Single- and Dual-Task variables in the MCI Group

	TUGT [s]	TUGT_**MAN**_ [s]	TUGT_**COG**_ [s]	DTC_**MAN**_ [%]	DTC_**COG**_ [%]	TUGT* vs TUGT_**MAN**_ [s]	TUGT** vs TUGT_**COG**_ [s]
**MMSE** [pts.]	−.12	−.11	−.13	−.09	−.07	.12	.16
**10MWT normal speed** [m/s]	−.59***	−.57***	−.52***	−.17	−.12	.31***	.32***
**10MWT** **fast speed** [m/s]	−.60***	−.58***	−.52***	−.17	−.12	.32***	.33***
**FES-I** [pts.]	.26	**.33**	.28	.07	.08	.15	.20
**30sChS** [x stand up]	−.62***	−.55***	−.54***	−.27	−.15	.38***	.35***
SLS OP* [s]	−.37***	−.45***	−.39***	−.00	−.07	.12	.21
SLS CL* [s]	−.32***	−.46***	−.40***	.07	.03	.08	.13
**TUGT** [s]	1.00	.85***	.82***	.53***	**.33**	−.70***	−.61***
**TUGT**_**MAN**_ [s]	.85***	1.00	.91***	.10	.01	−.33***	−.32***
**TUGT**_**COG**_ [s]	.82***	.91***	1.00	.20	−.15	−.39***	−.17

**Abbreviations:** *Differences between gait speed median changes under single-task TUGT and dual-task TUGT Manual conditions, ** Differences between gait speed median changes under single-task TUGT and dual-task TUGT Cognitive conditions ***p < 0.05

### DTC and functional outcomes

The analysis revealed a moderate, positive and statistically significant correlation between the DTC of motor-motor tasks (TUGT_MAN_) and single task TUGT.

There was a weak, statistically significant correlation between the DTC of motor-cognitive tasks TUGT.

An adjusted, cross-sectional model was developed to demonstrate the effect of the TUGT alone on the outcome of the dependent variable DTC_MAN_ in the subjects affected by MCI.

Around 25% of the variance of the DTC_MAN_ result is accounted for by gait performance in the single-task conditions (TUGT). In the case of Dual Task Cost Cognitive (DTC_COG_), this value is equal to ca. 10%. A 1% change in DTC_MAN_ corresponds to a ca. 0.5 s change in TUGT, while a 1% change in DTC_COG_ entails a ca. 0.35 s change in TUGT walking time. Independent predictors of DTC_MAN_ are outlined in Table 4.

**Table 4:** Independent predictors of DTC_MAN_

Predictor	DTC_**MAN**_ (%)		
	*R*^*2*^ *= 0.25 P = 0.001*		
	B^**a**^	Beta^**b**^	P-Value^**c**^
**TUG** [s]	0.54	0.53	0.001

**Abbreviations:** B^a -^ Unistandardized coefficients, Beta^b^- Standard coefficients, P-Value^c^ - Significant value, R^2−^Coefficient of determination

### Changes in gait speed between single-task and dual-task conditions

Under both types of dual-task conditions (TUGT_MAN_ and TUGT_COG_), there was a weak correlation between both normal and fast gait speed during the 10MWT, the strength of the lower limbs (30sChS), and gait performance under the single-task conditions.

## Discussion

The results pertaining to functional capacity of the institutionalized older adults affected by MCI correlated with their performance in the dual-task, motor-motor (TUGT_MAN_), and motor-cognitive (TUGT_COG_) activities. The most significant dependences were evident with regard to gait speed (including both normal speed and the fastest speed the subjects were able to attain without putting themselves at a considerable risk of falling), and the strength of the lower limbs assessed by the 30sChS test. The highest decrease in gait speed, i.e. 6.9%, was found in the subjects examined by the TUGT_MAN_ test. Doi et al. investigated the variations in the performance of the dual-task activities and the gait speed observed among the subjects affected by different forms of MCI [22]. Mean decrease in gait speed, while performing an additional task, was 9%.

Doi et al. also demonstrated that gait speed was correlated with working memory, and that visual memory affected both the single-task and the dual-task gait performance, especially in the individuals with amnestic MCI diagnosed against the revised Peterson criteria [23, 24].

The subjects who participated in the present study lost points in the cognitive function test, mostly due to the problems specific to the working and episodic memory, which may give some grounds to believe that these are likely to be the predominant functions related to a reduced gait speed. Aside from memory impairment, overall complexity of the problem was also likely to adversely affect the gait speed, which implies that cognitive load appreciably affected these activities [25].

It is then hardly surprising that – when the TUGT_COG_ dual-task entailed reverse counting via the subtraction of 7 from a specific number – gait speed decreased by another 6%, as compared to the one during TUGT_MAN_. In relation to the single-task test values (TUGT), the respective decreases were 17 and 24%, thereby increasing the corresponding DTC values to 24% in the case of TUGT_MAN_ and to 29.44% in the case of TUGT_COG_. The DTC of a secondary task involving reverse counting, as reported by Montero-Odasso et al. for the individuals affected by MCI, was approx. 20%, despite the fact that in this case the subtracted number was 7, which presumably should have resulted in a higher cost. The 9% difference between our own result and the one obtained by Montero-Odasso might be attributable to the 7-point difference between the mean MMSE scores (21 and 28 points) [25].

Individuals affected by MCI exhibited a significantly decreased gait speed during the dual-task (DT) motor-cognitive activities. Interestingly enough, the DT motor-motor activities performed by the MCI subjects while walking seemed to attest to their impaired cognitive abilities. In the present study, 25.7% of the variance of the TUGT_MAN_ DTC result was explained by the TUGT (single-task test) result.

Furthermore, Tseng et al. noted that older adults affected by amnestic MCI exhibited a 14% reduction in gait speed during the dual-task activities; in comparison, the mean reduction in gait speed in a group of older adults characterised by normal cognition was 5% [26]. This gives some grounds to believe that the dual-task tests should actually be applied to assess the gait speed effectively.

It should also be highlighted at this juncture that approx. 10% of the study participants walked at a faster pace during the motor-cognitive tasks, disregarding it entirely. When conducting investigations pertaining to dual-task activities in the individuals affected by MCI, it should be assumed that 10% of the subjects disregard the secondary task in favour of completing the primary one, i.e. maintaining balance [5]. This may likely be related to the volume of the prefrontal cortex. Larger cortex volume correlated with higher gait speed during both single-task and dual-task activities, as well as a decreased variance of stride time during the single-task tests.

Besides, the risk of major metabolic disorders in neurotransmitter relations was 63% in the individuals with significantly decreased gait speed. The likelihood of lower cortex volume in the subjects who achieved average results in the dual-task activities was twice as high. Both the motor cortex and neurotransmitters are involved in the dual-task activities. It should also be noted that when the dual-task activities are performed, the amount of oxyhemoglobin reaching the prefrontal lobe increases, which may be a protective factor in the course of cortical volume loss [26].

There are few studies focused on evaluating functional activity in a broader context. It has clearly been demonstrated that strength of the lower limbs has an appreciable effect on the dual-task performance, as it correlates with the difference in the median results of both TUGT and TUGT_MAN_, as well as TUGT and TUGT_COG_, test pairs. These conclusions are further corroborated by other studies. Casas-Herrero et al. reported a correlation between functional capacity, incidents of falling, muscle mass, muscle strength, and muscle power in the individuals with the frailty syndrome, and concomitant frailty syndrome and MCI [27].

The present Authors established a negative correlation between the strength of the flexor and extensor muscles of the knee (− 0.83, *p* = 0.034 and − 0.73 *p* = 0.021) and the results of the TUGT test, including the version with the reverse counting. Performance in the dual-task activities in TUGT_MAN_ and TUGT_COG_ tests thus depends not only on the memory function and the prefrontal cortex volume, but also on the strength of the lower limbs. Sosnoff expanded the list of relevant factors by age, extent of disability, and gait characteristics, which accounted for 17% of the variance of DTC [28].

Whereas in the gait characteristics at issue, as yielded by TUGT, this accounted for approx. 25% of variance in the motor task cost in the model standardised against the select sociodemographic variables.

Finally, it is well-worth highlighting that making (regular) use of the dual-task procedures proves specifically beneficial not only to the individuals affected by MCI, but also to any persons affected by intellectual disability [29].

Further studies on MCI should also take into account the strategies applied by individuals, when increasing and reducing the gait speed during pursuit of dual-task activities. Pertinent reference values for gait speed in the MCI-affected individuals should best be established as a conclusive marker of cognitive dysfunction and impaired functional capacity.

## Conclusions

Functional capacity affected the dual-task performance, especially with regard to the motor-motor tasks.

DTC in the individuals affected by MCI was significantly reduced, being more dependent on the gait speed in the motor-motor tasks, which entail visual memory, than in the motor-cognitive tasks.

## Data Availability

The datasets generated and/or analysed during the current study are not publicly available due to institutional restrictions, although they might be made available by the Corresponding Author upon a reasonable request.

## Abbreviations

MCI: Mild cognitive impairment
ADL: Activities of Daily Living
MCR: Motoric Cognitive Risk
MMSE: Mini–Mental State Examination
TUGT: Timed Up and Go Test
10MWT: 10 Meter Walk Test
TUGT_MAN_: Timed Up and Go Manual
TUGT_COG_: Timed Up and Go Cognitive
DTC: Dual Task Cost
DTC_MAN_: Dual Task Cost Manual
DTC_COG_: Dual Task Cost Cognitive
FOF: Fear of Falling
FES-I: Falls Efficacy Scale International Version
30sChS: 30-s Chair Stand Test
2-Min Step: 2 Minute Step test
SLS: The Single Leg Stance
SLS OP: The Single Leg Stance Open Eyes
SLS CL: The Single Leg Stance Closed Eyes
DT: dual task
